# Physician experiences with sodium-glucose cotransporter (SGLT2) inhibitors, a new class of medications in type 2 diabetes, and adverse effects

**DOI:** 10.1017/S1463423618000476

**Published:** 2018-07-23

**Authors:** Laura Patakfalvi, Anne-Sophie Brazeau, Kaberi Dasgupta

**Affiliations:** 1 Department of Family Medicine, McGill University, Montréal, Québec, Canada; 2 Department of Medicine, Research Institute of the McGill University Health Centre, Montréal, Québec, Canada

**Keywords:** diabetes drugs, diabetic ketoacidosis, SGLT2 inhibitor, sodium-glucose cotransporter inhibitor, type 2 diabetes

## Abstract

**Aim:**

The primary aim of our study is to identify physicians who have witnessed a complication attributed to sodium-glucose cotransporter (SGLT2) inhibitors. The secondary aim is to determine the type, severity, and setting of the event (inpatient versus outpatient).

**Background:**

Diabetes is an increasing public health burden with 9.9% of Canadians expected to be diagnosed with it in 2020. A prominent change with respect to treatment options since the publication of the revised Diabetes Canada guidelines in May 2016 concerned the SGLT2 inhibitors. Their favorable clinical profile has increased interest among clinicians, but there is still reason for caution. Because these drugs are new, the balance of benefits versus risks is not well understood.

**Methods:**

We conducted a cross-sectional survey of all in-practice physicians (excluding pediatricians). Data were collected through an online survey.

**Findings:**

Our survey identified 154 physicians who have identified one or more adverse drug reactions (ADRs) related to SGLT2 inhibitor use. A total of 173 ADRs were identified. In total, 20.6% of family physician respondents had witnessed one or more ADRs. The most common complication is mycotic infection (82 cases) with 47% identified as a low level of severity and occurring mostly in the outpatient setting. The second most common complication is diabetic ketoacidosis (43 cases) with 67% identified as a high level of severity and occurring mostly in the inpatient setting. Other identified complications include hyperkalemia (6 cases), renal insufficiency (15 cases), and even amputation (2 cases). Our survey is the first to document real-world complications from SGLT2 inhibitors. In the outpatient setting, mycotic infections are most common and most often benign. In the inpatient setting, diabetic ketoacidosis is the most common and is severe. This is an important take-home message for family physicians to tailor their practice and vigilance according to the practice setting.

## Background

Diabetes is an increasing public health burden with a predicted 10% of Canadians expected to be diagnosed by 2020 (Canadian Diabetes Association, 2011). With the increasing burden of disease and the scarce resources in health care, the Canadian Diabetes Association recommends integrating the Chronic Care Model for diabetes management. This organizational approach shifts the management of diabetes from specialist based on a multidisciplinary approach with the primary care physician at the forefront (Robinson *et al*., 2013). This puts primary care physicians at the forefront of diabetes management, both in inpatient and outpatient settings. More than 20% of diabetics will not see an endocrinologist, and this number will continue to rise (Saudek, 2002).

Several antihyperglycemic therapies have emerged in the past decade, with each new therapy influencing the prescribing patterns of physicians (Sharma *et al*., 2016). The newest class is the sodium-glucose cotransporter (SGLT2) inhibitors, including canagliflozin, dapagliflozin, and empagliflozin. These agents operate at the proximal renal tubule by inhibiting glucose and sodium reabsorption and thus increasing their excretion in the urine. They not only reduce glucose levels but also lower blood pressure.

An update to the Diabetes Canada guidelines includes the option of adding SGLT2 inhibitors to metformin or to a sulfonylurea plus metformin if glycemic targets are not met (Goldenberg *et al*., 2016). In addition to glucose and blood pressure lowering, these agents have the added benefits of weight loss and some lipid-lowering effects (Booth *et al*., 2016). The *Empagliflozin, Cardiovascular Outcomes, and Mortality in Type 2 Diabetes* trial (EMPA-REG trial) was the first diabetes drug trial showing that a member of the SGLT2 inhibitor class, empagliflozin, not only reduced glucose levels but also improved cardiovascular outcomes in patients with type 2 diabetes, and decreased mortality from all causes (Zinman *et al*., 2016). The *Canagliflozin and Cardiovascular and Renal Events in Type 2 Diabetes* trial confirmed the SGLT2 inhibitor canagliflozin had cardiovascular and renal benefits (Neal *et al*., 2017). However, the trial did demonstrate an approximate doubling of lower-limb amputation risk.

As for all therapies, benefits and risks must be understood and considered. Because these drugs are new and the duration of studies available is limited, the balance of benefits versus risks is not yet completely understood. While these agents have certainly demonstrated favorable outcomes, clinical trials demonstrate increased lower-limb amputation (for canagliflozin), higher rates of urogenital infections, and in some cases symptomatic postural hypotension, particularly in combination with diuretic therapy. In May 2014, canagliflozin was the first agent of its class to be approved for use by Health Canada for lowering blood glucose in adults with type 2 diabetes. Health Canada approval followed for dapagliflozin (December 2014) and empagliflozin (July 2015). At the time of publication, it has been two years since these agents have been approved in Canada with an increase in prescriptions since reporting of the cardiorenal benefits observed in the EMPA-REG trial (Zinman *et al*., 2016).

In May 2016, Health Canada issued a warning about the potential serious side effect of diabetic ketoacidosis (DKA), with five Canadian cases and 419 international cases reported. This adverse effect had not been apparent in clinical trials. While DKA is often associated with type 1 diabetes, it is unusual in type 2 diabetes. An even rarer entity, euglycemic DKA, defined as DKA without hyperglycemia, has been reported in case reports and case series of patients treated with SGLT2 inhibitors. These are serious and potentially fatal complications (Rosenstock and Ferrannini, 2015).

Real-world data about SGLT2 inhibitors are limited in the medical literature and the guidelines on their recommended use are based on clinical trials. Increased physician awareness of the potential adverse drug events (ADR) associated with SGLT2 inhibitors and their severity will allow better-informed treatment decisions and allow their timely identification and management. The present study endeavored to gain some understanding of the types of ADRs observed with SGLT2 inhibitor use in real-world settings through a survey of Canadian physicians.

## Methods

We conducted an online/paper survey in a convenience sample of fully licensed physicians with adult patients. Procedures were approved by the Institutional Review Board of the McGill University Health Centre (MUHC). The MUHC Research Ethics Board (REB), more precisely its Cells, Tissues, Genetics & Qualitative research panel (CTGQ) provided approval on 9 May 2017 (2017/3391). The following documents were approved or acknowledged: initial submission form (F11NIR-15269), REB conditions & PI Responses Forms (F20-16860, F20-18010), which include the research protocol, the consent form, the questionnaire and recruitment document in English and in French.

### Recruitment

Physicians were recruited during announcements at clinical rounds at McGill University-affiliated institutions and physician social media platforms (Facebook). We sent direct email invitations to individual physicians working in family medicine, internal medicine, and endocrinology clinics affiliated with McGill University, as well as some direct contacts at other institutions in Montreal and across Canada. Fully licensed physicians with adult patients were included in the study. Physicians who only treated pediatric patients and those who were not licensed for independent practice (medical students and residents) were excluded.

We cannot estimate the number of physicians who were aware of the survey. We posted information about the survey on social media, advertised it at various clinical rounds, and distributed it through contacts by email lists but we did not have access to the complete mailing lists ourselves as these were confidential.

### Questionnaire

The questions were designed by the authors through an iterative process. Five colleagues that were thought to represent the target respondents (from different specialties and different number of years in practice) reviewed the questionnaire independently and suggested modifications. Modifications included changes in wording, and the addition of certain open-ended questions. The survey was provided in English and in French, and was mainly in the form of multiple-choice questions with options for comments to be added if needed. Physicians willing to participate contacted us by email and were forwarded a link to access the online survey (Fluidsurveys™). In some instances, a paper copy was provided. Participants were entered into a lottery for a fitness tracker.

Demographic data on physician respondents were obtained including sex, number of years in practice, and field of practice. The survey asked physicians about whether they had ever observed an ADR that they believed to be related to SGLT2 inhibitor use. Each questionnaire included a minimum of four questions and three additional questions for each reported ADR.

If the respondent reported having observed at least one ADR, he/she was asked about the number of patients observed with SGLT2 inhibitor use-related ADRs. For each ADR, its nature, the setting in which it was witnessed (inpatient or outpatient) as well as its severity was queried (see Appendix for questionnaire). If more than 10 ADRs were witnessed, the survey respondents were asked to email the authors for further instructions, as the Fluidsurvey was programmed to allow for descriptions of 10 ADRs.

Severity was classified using an adapted version of ADR severity assessment scale developed by the Hartwig *et al*. (1992). The original scale includes seven levels of severity, ranging from no change in therapy (level one), discontinuation of therapy without other antidote (level two), discontinuation of therapy with antidote or other treatment (level three), ADR requiring at least one day of hospitalization (level four), ADR requiring intensive medical care (level five), ADR causing permanent harm (level six), and ADR leading to death (level seven). Levels one to two are classified as mild, levels three and four are moderate, and levels five to seven are severe. We adapted the scale (Table 1) to be more reflective of approaches to SGLT2 adverse reaction management.

**Table 1:** Sodium-glucose cotransporter (SGLT2) inhibitor-attributed adverse drug reactions (ADR) severity assessment scale

Level of severity	Criteria
Very mild	ADR requires treatment but no cessation of SGLT2
Mild	ADR requires cessation of the SGLT2 with or without treatment
Moderate	ADR requires leads to hospital visit or increase in hospital stay if
Severe	ADR requires intensive medical care
Very severe	ADR lead to permanent disability
Lethal	ADR is lethal

Analysis SPSS statistical software (version 24, 2016) was used for analysis. For field of practice, respondents who selected both family medicine and emergency medicine were classified under emergency medicine. Respondents who selected family medicine and another category (eg, palliative care) were classified under family medicine. The authors categorized each SGLT2 inhibitor-attributed ADR reported into one of the following categories: urogenital infections, hyperkalemia, renal insufficiency, or DKA. If the description of the ADR did not fit into any of these categories, it was classified under ‘other.’ Categorization of responses was conducted by two authors (L.P., A.S.B.) and disagreements were resolved through discussion among all three authors. If respondents selected more than one severity level for a given ADR, the most severe level was retained for analysis. Emergency room visits were classified as hospitalizations. We computed numbers and proportions for physician characteristics (province, sex, age categories). We calculated numbers and proportions with 95% confidence intervals (CIs) for categories of numbers of patients with ADRs observed, physicians within a category observing at least one ADR, types of ADRs, and severity categories of ADRs by types.

## Findings

Overall, 163 physicians completed the survey between May 17 and 18 July 2017. In total, 22 surveys were excluded (one completed by a pediatrician; 21 incomplete). >From the 21 incomplete surveys, three respondents had reported more than 10 ADRs but did not email us to receive further instruction. No data were gathered concerning these ADRs. Ten of the 21 surveys did contain partial information about number of ADRs witnessed but did not qualify each ADR, and were excluded. Survey respondents were largely from Quebec (88.7%). Slightly more than half were women (54.2%). Respondents were roughly equally distributed across categories of years of practice (38.0%<five years; 33.1% 5–20 years; 28.9%>20 years). Among the 142 respondents, 34.5% of the respondents had seen at least one patient with an SGLT2-inhibitor-related complication (52% were female, 95% were from Quebec, 40% had less than five years of practice, and 60% were family physicians). There was a similar distribution among the respondents who had not witnessed an SGLT2-inhibitor-related complication (55% female, 97% from Quebec, 43% with less than five years of practice, and 55% were family physicians).

Among the 142 respondents in whom data were examined, 49 (34.5%, 95% CI 26.7–42.4%) reported having witnessed at least one patient with an SGLT2 inhibitor-attributed ADR. Among these, 14 witnessed one (28.6%, 95% CI 15.8–41.4%), 14 witnessed two (28.6%, 95% CI 15.8–41.4%), and the remainder had witnessed between 3 and 21 (42.8%, 95% CI 28.9–56.9%). The proportion who had witnessed at least one ADR were 20.6% (95% CI 10.7–30.5%) of family physician respondents (14/68), 54.6% (95% CI 19.5–89.6%) of emergency room physicians (6/11), 56.5% (95% CI 34.6–78.4%) of internists (13/23), and 81.3% (95% CI 59.8–102.7%) of endocrinologists (13/16).

A total of 173 ADRs were reported across 165 patients. The most common complication witnessed was mycotic infection (82 cases, 47%) followed by DKA (43 cases, 25%), renal insufficiency (15 cases, 9%), and hyperkalemia (six cases, 3%). In total, 27 other ADRs were reported, the most common of which was urinary infection (seven cases, 4%), followed by urinary symptoms (six cases, 3%). Two cases of amputation were reported (1%) (Table 2).

**Table 2:** Sodium-glucose cotransporter inhibitor-attributed adverse drug reactions (ADR)

Type of ADR	Number of ADR
Mycotic infection	82
DKA	43
Renal insufficiency	15
Hyperkalemia	6
Others	
Urinary infection	7
Urinary symptoms	6
Amputation	2
Severe weight loss	2
Thirst/dehydration	2
Fall	1
Diarrhea	1
Foot ulcer	1
Rash	1
Visual symptoms	1
Hypoglycemia	1
Hospitalization	2

DKA=diabetic ketoacidosis.

For mycotic infection, among the 82 ADRs, 48 (58.5%, 95% CI 47.8–69.3%) were treated without discontinuing the agent (very mild severity), 28 (34.1%, 95% CI 23.8–44.5%) led to discontinuation of the drug and six to hospitalization or increased length of hospital stay (7.3%, 95% CI 1.6–13.0%). The majority were observed and managed in the outpatient setting (76/82, 92.7%, 95% CI 87.0–98.4%). For DKA, among the 43 ADRs, four were mild (9.3%, 95% CI 0.5–18.1%), 29 were moderate (67.4%, 95% CI 53.3–81.6%), and 10 were severe (23.3%, 95% CI 10.5–36.0%). There were no complications that led to permanent harm (very severe) or death (lethal). The majority were observed and managed in the inpatient setting (42/43, 97.7%, 95% CI 93.1–102.2%) (Table 3).

**Table 3:** Cases of diabetic ketoacidosis (DKA) and mycotic infection according to severity and location

	Complication					
Level of severity	DKA			Mycotic infection		
	Inpatient	Outpatient	Total	Inpatient	Outpatient	Total
Very mild	0	0	0	3	45	48
Mild	4	0	4	0	28	28
Moderate	28	1	29	3	3	6
Severe	10	0	10	0	0	0

## Discussion

To our knowledge, our survey is the first to document real-world complications from SGLT2 inhibitors. It suggests that witnessing an ADR attributed to SGLT2 inhibitor use is common with over one-third of physician respondents reporting witnessing at least one patient with one or more ADRs.

Primary care physicians are faced with challenges in diabetes management. In general, in terms of overall practice patterns, it has been shown that specialists practicing in their area of expertise are more likely to use medications associated with improved survival (Harrold *et al*., 1999). New medications are often used by specialists until experience is gained. However given the potential benefits of SGLT2 inhibitors, it is important for primary care physicians to know the potential side effects in order to better integrate SGLT2 inhibitors into their therapeutic approach.

Our study revealed that 39% of all physicians and 20% of family physician respondents had witnessed at least one patient with an ADR. Overall, the most were mycotic infection (47%), followed by DKA (25%).

The types and severity of the ADRs are closely correlated with the setting in which they are witnessed (inpatient versus outpatient). This finding is especially important for general practitioners who tailor their practice according to the practice setting and the expected prevalence of ADR. Inpatient family physicians should closely monitor for DKA, while those practicing in outpatient clinics should monitor for mycotic infection.

The most common SGLT2 inhibitor-associated ADR is mycotic infection with a total of 80 witnessed among 142 physicians surveyed. We do not know what proportion this represents among the total number of SGLT2 treated patients across these 142 physicians. The incidence of mycotic infections in SGLT2 treated patients in clinical trials is 4–6% (Chaplin, 2016). In our study, more than half of the mycotic infections (59%) were treated without discontinuing the SGLT2 inhibitor. Primary care physicians are faced with the challenge of managing SGLT2-associated mycotic infections as there are no formal guidelines. These usually occur within the first four months of SGLT2 inhibitor treatment but first episodes can occur up to a year into treatment (Johnsson *et al*., 2013). Certain risk factors for mycotic infections have been identified, which include female sex, being an uncircumcised male and previous history of mycotic infection (Chaplin, 2016).

A pooled analysis of clinical studies with dapagliflozin (Johnsson *et al*., 2013) demonstrated that those with a prior history of recurrent mycotic infections develop them more frequently when on dapagliflozin compared with placebo. When cultures are obtained, the identified pathogen is most commonly *Candida*, which resolves spontaneously or responds to conventional topical or oral agents (Johnsson *et al*., 2013). Most mycotic infections are mild and if treated, resolve with one course of treatment. In two separate reviews of clinical studies with canagliflozin (Nyirjesy *et al*., 2014) and dapagliglozin (Johnsson *et al*., 2013), recurrent infections were uncommon. In those treated with canagliflozin, among those who developed a mycotic infection, 80% only reported one episode. Women with a prior history of recurrent mycotic infections were 2.5 times more likely to experience one when being treated with canagliflozin. Given the current evidence, we would suggest that the potential risk for mycotic infection should not deter a primary care physician from choosing an SGLT2 inhibitor. If recurrent mycotic infections develop, the decision whether to continue with the SGLT2 inhibitor should be discussed with the patient.

The second most common SGLT2 inhibitor-associated ADR is DKA. Over 90% of DKA ADRs led to hospitalization or extended length of stay in already hospitalized patients. In total, 44 cases of DKA were reported in this study, which exceeds the number published in a recent systematic review that enumerated case reports worldwide (34 case reports) (Burke *et al*., 2017).

Although DKA in general is a complication of type 1 rather than type 2 diabetes, acute medical illnesses and trauma may lead to DKA in some type 2 diabetes patients. Although DKA was not reported in the initial SGLT2 inhibitor trials (Zinman *et al*., 2016; Neal *et al*., 2017), in 2015, based on numerous adverse event reports, Health Canada issued statements that SGLT2 inhibitors may be associated with an increased risk of DKA. A total of 34 worldwide case reports have been identified in a recent systematic review (Burke *et al*., 2017). According to Health Canada’s latest report, a total of five DKA cases associated with SGLT2-inhibitor use have been reported in Canada (Government of Canada, 2016). The number of cases reported in our review exceed both these numbers. This strongly suggests that the true incidence of DKA is higher than currently appreciated and that not all clinicians publish or report their DKA cases.

Our study also highlights the fact that DKA is most frequently seen in the inpatient setting. This is an important take-home message, especially for primary care physicians who work in multiple care settings. There are no current formal guidelines for SGLT2 inhibitor use in hospitalized patients. The potential benefits of both continuing and starting SGLT2 inhibitor therapy in the inpatient setting include decreased risk of hypoglycemia and improved treatment of heart failure and hypertension. However, it is well known that several characteristics of the hospitalized patient including infection and fasting increase the risk of SGLT2 inhibitor-associated DKA (Levine *et al*., 2017). In euglycemic DKA, the triad of laboratory findings leading to the diagnosis of DKA (hyperglycemia>14 mmol/L, serum bicarbonate⩽18 mEq/L, and pH⩽7.4) is often absent. This leads to delays in identification and treatment of euglycemic DKA. The overall mortality rate of DKA is between 0.2 and 2%. Levine *et al*. have discussed the importance of early identification of euglycemic DKA through the implementation of an automated alert system where an alert would occur if patients on SGLT2 inhibitors has a bicarbonate<18 mEq/L and anion gap>12. However, real-world data testing this approach are not available. Therefore, we would suggest that primary care physicians in the office avoid prescribing SGLT2 inhibitors for patients who are frail or frequently hospitalized and therefore more at risk for hypoglycemia. We suggest that primary care physicians in the hospital setting weigh the risks and the benefits related to their decision to continue or temporarily stop SGLT2 inhibitor therapy.

Our study had several limitations. Our sample represents a convenience sample that did not involve systematic sampling of physicians across Canada. A selection bias might have been introduced given the fact that doctors who had not observed SGLT2 inhibitor-associated ADRs may have been less inclined to participate. Physicians were asked to list ADRs that they believed to be related to SGLT2 inhibitor use. This is a subjective measure and the validity of the ADR attributions were not verified. The survey was not designed to collect data on individual SGLT2 inhibitors or on timing of the ADR relative to the start of the treatment. Future studies should aim to analyze each SGLT2 individually, as well as clarify the timing of the ADR in regard to the start of treatment.

## Conclusion

With the use of SGLT2 inhibitors on the rise, it is important to gather real-world data. This will enable clinicians to better tailor their practice and become vigilant to reduce future events. This finding is important for family physicians who practice in a variety of health care settings. In the outpatient setting, mycotic infections are most common and most often benign. Discontinuing the agent with the occurrence of a mycotic infection may not be necessary but specific management trials are warranted given the likelihood of increased use of this class of agents in clinical practice. Our study revealed more cases of DKA than those identified in a recent systematic review (Burke *et al*., 2017). This highlights the fact the prevalence of DKA might be higher in the real-world setting compared with the trials. Population-level postmarketing surveillance is warranted. If a decision is made to use SGLT2 inhibitors in the inpatient setting, vigilance is required to monitor for the occurrence of DKA.

Nonetheless, SGLT2 inhibitors are a novel and promising drug class. Primary care physicians should use individualized and informed treatment decision‐making to determine if a patient is a good candidate to start this medication. The decision to continue the SGLT2 inhibitor should be re-evaluated at each outpatient visit and in the event of a hospital admission.

## Appendix

**Questionnaire**

**Physicians’ perspective on SGLT2 inhibitors**

1. In which country do you practice?
  - Canada (chose province)
  - Other (please specify)
2. What is your gender?
  - Male
  - Female
  - Other
3. In what field of medicine do you practice? (you can select more than one option)
  - Family medicine
  - Emergency medicine
  - General Internal medicine
  - Endocrinology
  - Other (please specify)
4. How many years have you been in practice (after residency and/or fellowship training)?
  - <5
  - 5–20
  - 20 or more
5. Have you witnessed any adverse events associated with SGLT2 use? YES or NO (EXIT option)
6. How many adverse events have you witnessed? (According to the number of cases witnessed, questions 7, 8, and 9 will ask the same question for each case)
7. For adverse event noted in patient 1, I have witnessed the following complications:
  - Diabetic ketoacidosis
  - Mycotic infections
  - Hyperkalemia and renal insufficiency
  - Other (Describe other complications that you attribute to SGLT2 inhibitors seen in patient 1)
8. For adverse event noted in patient 1, which setting was the event witnessed in?
  - Hospital
  - Long-term care center
  - Outpatient clinic
9. For adverse event noted in patient 1, how severe was the adverse event? (Choose all that apply)
  - The event required that the SGLT2 inhibitor be withheld, discontinued, or changed. No other treatment was required.
  - The event did require treatment, but no cessation of SGLT2 inhibitor. Please indicate what treatment:
  - The event lead to hospital visit or admission or increase in length of stay if patient was already hospitalized.
  - The event led to intensive medical care
  - The event was lethal.

**Questionnaire**

**Perspective des médecins sur les inhibiteurs de SGLT2**

1. Dans quel pays pratiquez-vous?
  - Canada
  - Autre (veuillez préciser)
2. Quel est votre sexe?
  - Homme
  - Femme
  - Autre
3. Dans quel domaine de la médecine pratiquez-vous la médecine? (Vous pouvez sélectionner plus d'une option)
  - Médecine de famille
  - Médecine d'urgence
  - Médecine interne générale
  - Endocrinologie
  - Autre (veuillez préciser)
4. Combien d'années avez-vous été en pratique (après la résidence et / ou la formation de fellowship)?
  - <5
  - 5–20
  - 20 ou plus
5. Avez-vous été témoin à des événements indésirables associés à l'utilisation de SGLT2? OUI ou NON (option EXIT)
6. Combien d'événements indésirables avez-vous vus? (Selon le nombre de cas, les questions 7, 8 et 9 poseront la même question pour chaque cas)
7. Pour l’événement indésirable vu dans patient 1, j'ai été témoin des complications suivantes:
  - Acidocétose diabétique
  - Infections mycotiques
  - Hyperkaliémie et insuffisance rénale
  - Autre (Décrivez les autres complications que vous attribuez aux inhibiteurs de SGLT2 vu dans votre patient 1)
8. Pour le patient 1, dans quel contexte avez-vous été témoin de l'événement indésirable?
  - Hôpital
  - Centre de soins de longue durée
  - Clinique ambulatoire
9. Pour le patient 1, quelle est la gravité de l'événement indésirable? (Choisissez tout ce qui correspond)
  - L'événement a exigé que le traitment avec inhibiteur de SGLT2 soit retenu, interrompu ou modifié. Aucun autre traitement n'était requis.
  - L'événement nécessitait un traitement, mais pas de cessation de l'inhibiteur de SGLT2.
    Veuillez indiquer quel traitement:
  - L'événement a nécessité une visite ou une admission à l'hôpital ou une augmentation de la durée du séjour si le patient était déjà hospitalisé.
  - L'événement a nécessité des soins médicaux intensifs
  - L'événement a été mortel.
